# Field Trial of an Aerially-Distributed Tuberculosis Vaccine in a Low-Density Wildlife Population of Brushtail Possums (*Trichosurus vulpecula*)

**DOI:** 10.1371/journal.pone.0167144

**Published:** 2016-11-28

**Authors:** Graham Nugent, Ivor J. Yockney, E. Jackie Whitford, Martin L. Cross, Frank E. Aldwell, Bryce M. Buddle

**Affiliations:** 1 Landcare Research – Manaaki Whenua, Lincoln, New Zealand; 2 Centre for Innovation, University of Otago, Dunedin, New Zealand; 3 AgResearch, Hopkirk Institute, Palmerston North, New Zealand; CSIRO, AUSTRALIA

## Abstract

Oral-delivery *Mycobacterium bovis* bacillus Calmette-Guérin (BCG) vaccine in a lipid matrix has been shown to confer protection against *M*. *bovis* infection and reduce the severity of tuberculosis (TB) when fed to brushtail possums (*Trichosurus vulpecula*), the major wildlife vector of bovine TB in New Zealand. Here we demonstrate the feasibility of aerial delivery of this live vaccine in bait form to an *M*. *bovis*-infected wild possum population, and subsequently assess vaccine uptake and field efficacy. Pre-trial studies indicated a resident possum population at very low density (<0.6 possums/ha) at the field site, with a 5.1% prevalence of macroscopic TB lesions. Pilot studies indicated that flavoured lipid matrix baits in weather-proof sachets could be successfully sown aerially via helicopter and were palatable to, and likely to be consumed by, a majority of wild possums under free-choice conditions. Subsequently, sachet-held lipid baits containing live BCG vaccine were sown at 3 baits/ha over a 1360 ha area, equating to >5 baits available per possum. Blood sampling conducted two months later provided some evidence of vaccine uptake. A necropsy survey conducted one year later identified a lower prevalence of culture-confirmed *M*. *bovis* infection and/or gross TB lesions among adult possums in vaccinated areas (1.1% prevalence; 95% CI, 0–3.3%, n = 92) than in unvaccinated areas (5.6%; 0.7–10.5%, n = 89); P = 0.098. Although not statistically different, the 81% efficacy in protecting possums against natural infection calculated from these data is within the range of previous estimates of vaccine efficacy in trials where BCG vaccine was delivered manually. We conclude that, with further straightforward refinement to improve free-choice uptake, aerial delivery of oral BCG vaccine is likely to be effective in controlling TB in wild possums. We briefly discuss contexts in which this could potentially become an important complementary tool in achieving national eradication of TB from New Zealand wildlife.

## Introduction

Aerial delivery of vaccine-laden baits has been used in Europe [1,2] and in North America [3] to control viral diseases among wildlife, such as classical swine fever (in wild suids) and rabies (in mesocarnivores). In this paper we document the development and experimental field testing of aerial delivery of a bacterial vaccine (*Mycobacterium bovis* bacillus Calmette-Guerin; BCG) aimed at helping control bovine tuberculosis (TB) caused by virulent strains of *M*. *bovis* among wildlife in New Zealand. Bovine TB is a zoonotic disease that affects cattle world-wide. In developed countries, the risk to human health is diminished by pasteurisation of milk and slaughterhouse inspection to prevent affected meat from entering the food chain, combined with efforts to eliminate the disease from livestock [4]. However, efforts to eradicate bovine TB have been undermined in some countries by the establishment of self-sustaining *M*. *bovis* infection in one or more wildlife host species [5]. In New Zealand, the major wildlife maintenance host of TB is the introduced brushtail possum (*Trichosurus vulpecula*) [6].

Bovine TB in New Zealand probably originated from infected livestock imports, and appears to have become established in some possum populations in the 1960s, subsequently becoming endemic in wildlife over about 11 million ha (40% of New Zealand) over the ensuing 30–40 years [6,7]. Brushtail possums are now virtually ubiquitous, with typical densities of 2–10 possums per hectare in forest habitat (but sometimes exceeding 20/ha [8]). In TB endemic regions with high possum densities, disease prevalence among possums in unmanaged areas has been recorded at around 5–10% [6, 9], and during the height of wildlife infection rates in the early 1990s, spillover infection from possums saw annual cattle herd infection rates exceed 10% [10, 11]. Over the ensuing two decades, an extensive programme of possum lethal control by poisoning and trapping [6], combined with routine livestock disease surveillance and movement control measures [12], has seen the level of infection in livestock decline by over 95% [7,11] and the size of the wildlife TB-endemic area shrink by a quarter [13].

To date, only one country (Australia) has succeeded in nationally eradicating TB from wildlife maintenance hosts, through lethal depopulation or removal of feral cattle and buffalo (*Bubalus bubalis*) [14]. At a regional level, New Zealand has also eradicated TB from brushtail possums in some locations using lethal control [7]. This has been achieved across easily accessible farmland areas predominantly using ground-based trapping and poisoning, but more than a third of New Zealand’s TB endemic area is heavily forested, and/or steeply mountainous, and/or remote. Ground-based methods are difficult or prohibitively expensive in such areas [15], but conversely the alternative of aerial deployment of toxins is sometimes contentious among landowners and the public [16], even though brushtail possums are widely viewed as conservation pests. In general, the practice of intensive lethal control of wild animals for disease management is controversial, particularly where the wildlife host(s) are highly valued, as is the case with white-tailed deer (*Odocoileus virginianus*) in Michigan USA [17] and with badgers (*Meles meles*) in Europe [18]. Hence there has been, and is, an ongoing need to also evaluate non-lethal means to control TB in wildlife.

There has been a longstanding interest in vaccination for control of TB in wildlife species [19]. Under experimental conditions, oral vaccination with BCG has been shown to be efficacious in protecting possums, badgers, wild boar (*Sus scrofa*) and white-tailed deer against TB [20]. In New Zealand, feeding possums with live BCG in a lipid matrix has been shown to confer a significant reduction in pathology and bacterial load after artificial *M*. *bovis* challenge, both in captive possums [21–23] and in free-living possums [24,25]; and to reduce the incidence of *M*. *bovis* infection acquired by natural exposure among wild possums in a forested environment [26]. However, the vaccination method in all of these experimental studies is not suited to operational use, as it was achieved by direct feeding of captive or captured possums with a known amount of vaccine matrix—possums had no freedom of choice about whether to consume the vaccine, or how much. In addition, in a practical sense, capturing wild possums to vaccinate (and then release) them would never be considered as a management method in New Zealand (unlike the current approach practised in England and Wales with badgers [19,27]), given possums are classified as noxious pests. The need, therefore, is for some form of free-choice oral baiting that would enable landscape-scale delivery of vaccine at low cost.

Here we describe the development and field testing of an aerial vaccine delivery system, based on large-scale distribution from a helicopter of live BCG embedded within a lipid matrix, delivered in bait form. As far as we are aware, this is the first field trial using a widely-distributed, free-choice oral vaccine against TB in wildlife. This trial necessitated pilot-scale investigation of a number of field parameters related to bait delivery, prior to the vaccine trial itself. We aimed first, and primarily, to demonstrate the feasibility of aerial delivery of the vaccine and to confirm (or not) its free-choice uptake by possums, secondly to characterise the resident possum population and ensure TB was present in the study area, and thirdly (subject to levels of vaccine uptake) to evaluate the efficacy of the vaccine in reducing TB levels in the local possum population.

## Materials and Methods

### Overall design

The main field trial involved aerial deployment of vaccine baits over two ~1000 ha areas in the northern South Island high country (NSIHC), with subsequent assessment of vaccine efficacy through comparison of TB levels in possums between those two blocks and two similar unvaccinated blocks. Prior to the main field trial, resident wildlife was surveyed to assess possum abundance and to confirm the on-going presence of TB. Accordingly, precursor pilot trials of bait sowing, indicative removal and uptake rates were conducted to refine the delivery system and guide our choice of bait sowing rates. Because TB prevalence in possums in this area varies widely in space [28], the level of *M*. *bovis* infection in the four blocks was supplemented by artificial infection of resident possums.

### Study area characteristics, possum population and disease dynamics

The field trial was conducted between August 2011 and October 2012 on Muzzle Station and the Clarence Reserve in the NSIHC region (42°15’ S, 173°05’ E). This previously uncontrolled area was chosen primarily because it exemplified a remote area with rugged terrain in which ground-based possum control would be difficult. This steeply montane area of semi-arid habitat is sub-optimal for possums [29], so possums are sparsely distributed and densities are low (typically <1/ha [30]) with individuals ranging more widely than in more favourable forest habitat (typical home range size 15–25 ha [31]). We considered that this combination of factors would minimise the number of vaccine baits needed to expose all possums to the vaccine while providing a context in which we could attempt to assess the efficacy of a voluntary-uptake vaccine.

Despite the low possum density, the study area is endemic for TB, and at outset the resident possum population was expected to harbour *M*. *bovis* infection. Previous cross-sectional surveys in the vicinity had recorded TB prevalences of 1–3% in possums since the mid-2000s [28,32]. A detailed wildlife disease survey conducted in the study area 4 years prior to the present study recorded a mean prevalence of gross TB lesions among resident possums of 2.7% (n = 450 [28]). Prevalence of disease among wild pigs in the area, as a result of spillover infection from possums, often exceeds 60% [33,34].

**Fig 1:**
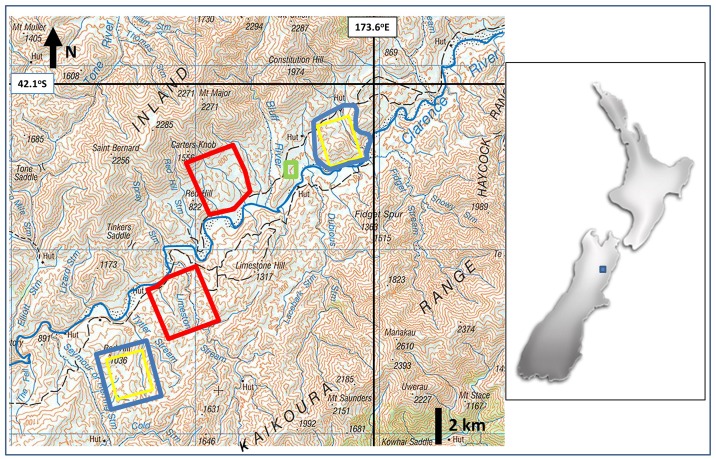
Location of the Muzzle Station/Clarence Reserve study sites within the northern South Island high country region of New Zealand. Four main study blocks are depicted in the regional-scale map (left) with vaccine blocks outlined in blue and non-treatment blocks in red. The yellow lines depict the inner 700 ha areas of the two vaccine blocks over which vaccine was deployed in September 2011. The green rectangle depicts the 50 ha block on which the pilot aerial baiting and bait contact studies were conducted.

Four main study blocks were established (Fig 1). Blocks averaged 3.8 ± 0.7 km from each other, with the closest linear distance of 0.85 km between two adjacent block perimeters (i.e. approximately twice the home range width of a typical possum home range area in this habitat [31]). Two blocks were randomly chosen for vaccination (972 and 999 ha), with the other two (981 and 991 ha) left unvaccinated (Fig 1). In the vaccine blocks, baits were distributed aerially over the central ~700 ha core area of the blocks (see below). For subsequent possum sampling, trapping/poisoning effort was focussed on favourable habitat within these cores, and/or on areas within the average possum home range radius (i.e. approximately 200-250m) of a vaccinated core if that area was bounded by a geographic barrier that prevented immigration of possums that might not have been exposed to vaccine (e.g. river). This was to reduce the possibility of sampling possums immigrating from outside of the vaccine-deployment area [35].

A 50 ha area used for the pilot sowing trial was established outside the main blocks (Fig 1).

### Ethics and environmental approval

All experimentation involving animals in this study was approved by the Landcare Research Animal Ethics Committee (approval number 10/09/02). Approval to conduct field work was granted by the Department of Conservation for work on public land (approval number NM-28859-FAU) and by the run-holder (Mr C. Nimmo, Muzzle Station, Kaikoura, New Zealand) for work on private land. Approval to conduct this work was also granted through liaison with the local iwi (indigenous tribe; Kaikoura Runanga) and via Environment Canterbury (Ecan) local government resource consent. Approval to conduct field work involving an unwanted organism (*M*. *bovis*) was granted by Biosecurity New Zealand under sections 52 and 53 of the Biosecurity Act. Permission to use a hazardous substance in the field (encapsulated cyanide pellets and cyanide-paste bait) was granted by the Medical Officer of Health for the Canterbury District Health Board, under HSNO approval no. 12/10/CHRPH/AH. The Environmental Risk Management Agency (ERMA), now the Environmental Protection Agency (EPA), granted permission for us to use a non-hazardous substance (lipid vaccine matrix) in response to a written enquiry from the vaccine manufacturer (dated 24/11/2008). Provisional registration of a vaccine containing live *M*. *bovis* BCG for field use in wildlife was obtained under the guidelines of the Agricultural Compounds & Veterinary Medicines register, and was approved by the NZ Food Safety Authority (approval number A010573).

### Pre-trial possum population characteristics, wildlife TB surveys and artificial infections

To obtain an estimate of possum abundance in the study areas, as a basis for determining vaccine sowing density and for estimating trial numbers, possums were trapped from the 50 ha pilot study site 11 months prior to commencement of the main trial. For this purpose, 184 baited leg-hold traps (Victor no. 1, Soft-Catch traps) were set over two consecutive nights along lines with 50 m trap spacings. Any possum captures were recorded and the animals were released. Catch rate data were entered into a population density estimator, as outlined by Ramsey et al. [36].

To confirm the likely broad-scale presence of TB in possums across the study area, leg-hold traps were set at ‘best-sign’ (i.e. habitat considered by trappers most favourable to possums) across the study area between the spring of 2009 and autumn 2010. Captured possums were euthanized and necropsied for TB following the procedure in the supplementary material (S1 File). At the same time, a sample of feral pigs (which are highly sensitive ‘sentinel host’ indicators of TB presence in possums [37]) was obtained from the study area by shooting, and these were also necropsied for TB diagnosis [32,33].

As the above survey and previous research [28] indicated a patchy spatial distribution of natural *M*. *bovis* infection among resident possums, it was uncertain that the background level of TB among the low density possum population within each block would be sufficient to allow statistically robust assessment of vaccine efficacy. Accordingly, we supplemented natural infection in the area with artificially-infected animals to ensure at least some *M*. *bovis* infection among the resident possums within each block. For this purpose, 54 possums (50 adults and 4 yearlings) were live-trapped at random sites across the study blocks in spring 2011, sedated, transported to a central location, and infected by sub-cutaneous injection of 20 colony-forming units (cfu) of virulent *M*. *bovis* strain 86/5701 into the webbing between the fore- and hind-limb digits of two feet, as described previously [38,39]. Possums were returned to their original capture locations within the study blocks and released. Vaccine-treatment blocks received 29 artificially-infected possums (13 females, 16 males) and non-vaccine blocks received 25 (12 females, 13 males).

### Pilot testing of aerial bait sowing, and assessment of bait removal and uptake

Pilot trials were undertaken to determine appropriate aerial bait sowing rates, and to determine likely encounter and consumption or removal rates of baits by possums or other wildlife. These pilot studies were all undertaken using the lipid vaccine-delivery matrix (without BCG) described by Aldwell *et al*. [21]. Briefly, these baits comprised 1 cc pellets of lipid matrix flavoured with 10% chocolate powder and 0.67% anise oil sealed in weatherproof sachets (Connovation Ltd, Auckland, New Zealand; as described previously in detail [40]).

Possum uptake of sachet-held baits was assessed by incorporating a marker dye into the lipid matrix (1% w/v rhodamine-B dye, as described previously [40]) and deploying 33 sachets at 20 m intervals along each of two transects within the 50 ha pilot-trial site. Chewcards (CC, a non-capture possum detection device [41]) were set at each site to identify the encounter rate by possums. Bait sachets were secured in position 30 cm above ground level using either metal spikes driven into the ground or by attaching the sachets to nearby vegetation, and were left in place for 3 consecutive nights, after which a flour-lured leg-hold trap was set at each site for two nights. Captured possums were euthanized and examined for rhodamine marking around the mouth, nose, paws and in the stomach contents.

In a second pilot trial, an aerial delivery system for sachet-held lipid baits was tested over the same 50 ha pilot site. A Robinson R-44 helicopter was flown at 22 knots approximately 65 m above the terrain, and sachet baits were dropped manually down a 1.3 m long/10 cm diameter delivery chute at 2.0 second intervals. We aimed to deploy one sachet bait every 23 m along five parallel 1 km-long flight paths spaced 100m apart (Table 1). Early the following day, field staff searched on foot along the flight paths and counted the sachets that were visible within line-of-sight. To assess the fate of aerially-deployed baits, field staff then set a grid pattern comprising ten 500 m-long transects spaced 100 m apart that were set at 90 degrees to the flight paths, and re-searched the area on foot later on the same day, recording the location of any deposited bait; any position with a sachet-held bait was marked as a waypoint and then revisited three days later to determine its fate, with any remaining lipid baits removed at that time. Next, to assess uptake of the lipid bait relative to that for a bait type known to be highly palatable to possums, commercially-produced baits were placed at 146 waypoint-marked locations along the same transects. These baits comprised a cinnamon-lured cereal matrix (Animal Control Products, Whanganui, New Zealand) deployed similarly in weatherproof sachets, and again their fate was determined after a further 3 nights.

**Table 1:** Overview of the flight characteristics during aerial sowing of flavoured lipid baits in sachets during the pilot trial and during the main vaccine trial (the latter containing live *M*. *bovis* BCG).

	Baiting and area characteristics			Bait deployment characteristics	
	Area	No. baits deployed	Target density	Aircraft speed	Flight line length/path spacing
Pilot trial	50 ha	186	4.4 flavoured lipid baits (no BCG)/ha	22 knots (1 bait/3.0s)	1.0 km lines, 100 m fps
Vaccine trial	1970 ha (pre-feed)	>30,000	175 flavoured cereal baits/ha	44 knots (broadcast sowing)	4 km lines, 150 m fps
	1360 ha (Main deployment)	4100	3.0 flavoured lipid baits (including BCG)/ha	30 knots (1 bait/3.0s)	2.6 km lines, 150 m fps
				35 knots (1 bait/2.5s)	

Fps, flight path spacing

In the final pilot trial, a single-choice bait removal study was conducted on the main study site over 7 nights. In the four trial blocks, 316 lipid sachets were placed on the ground, by hand, at 50 m intervals along thirty-two 500 m-long transects. As far as possible, they were placed out of direct wind and sun and held in place by a metal spike, with a CC also placed at each site to record proximate possum activity. The sites were checked after 7 nights to determine (from bite marks on the CCs) which sites had definitely been encountered by possums, and to determine whether the lipid bait had been eaten.

### Vaccine and vaccine deployment

Vaccine was supplied by Immune Solutions Ltd (Dunedin, New Zealand) in flavoured bait form. Briefly, this comprised an estimated 10^8^ cfu of live *M*. *bovis* BCG (Danish strain 1311) formulated into the edible lipid matrix described above; a previous field trial has shown this combination to be attractive and palatable to wild possums [40].

In an effort to familiarise possums with artificial bait, the main trial blocks were pre-fed with 2 g-size RS5 cereal possum baits (Pest Control Research Ltd, Christchurch, New Zealand) two weeks before vaccine delivery. Like the lipid baits, these cereal baits were lured with chocolate powder and anise but were not enclosed in sachets, and were sown using a helicopter-borne broadcast-sowing bucket [42] flown along eighteen 2.6 km-long parallel flight paths spaced 150 m apart, at a rate of 175 baits/ha. For vaccine deployment (September 2011), sachet-held baits were dropped (via delivery chute) from a helicopter flying along the same flight paths at low speed at 25–100 m above ground level. Flight path alignment was by GPS guidance and cruise speed was 30 knots (with 1 bait dropped per 3.0 seconds) or 35 knots (1 bait per 2.5 seconds) to deploy at a target density of 3 baits/ha (Table 1). This target sachet bait density was considered to be the best compromise between numbers of possums thought present and the possums’ known movement behaviour (home range size) in the NSIHC habitat, taking into account the high time and monetary cost of manually producing the ~4100 live vaccine baits required.

### Post-vaccination immune reactivity

In November 2011 (two months after vaccine deployment), leg-hold traps were set throughout the vaccine blocks and one of the non-vaccinated blocks. Captured possums were sedated and ear-tagged, and a 2 mL heparinised blood sample was drawn from the tail or jugular vein; possums were then released (all methods as described previously [24,26]). Blood samples were collected from 61 possums, transferred to a PC2-level laboratory and stored at 4°C overnight, prior to assay. A lymphocyte proliferation assay (LPA) was conducted to determine the cell-based immune reactivity to purified protein derivative of *M*. *bovis* (PPD-B), as described previously [22,43]. Briefly, blood was depleted of erythrocytes, mononuclear cells were cultured for 4 days with or without PPD-B, and proliferation of lymphocytes was measured by the uptake of tritiated thymidine added 18 h prior to the end of the incubation. Results were expressed as stimulation indexes (SI = mean counts per minute (cpm) from triplicate cell cultures stimulated with PPD-B divided by the mean cpm from triplicate cultures with phosphate-buffered saline). The cut-off for determining a positive response to PPD-B was any response exceeding the 99 percentile of the distribution of responses observed among possums from non-vaccinated areas, i.e. values > mean SI plus three standard deviations observed in controls [43].

### Efficacy against natural infection with *M*. *bovis*

In October 2012 (13 months after vaccine deployment) possums were culled from vaccine and non-vaccinated blocks, either by leg-hold trapping followed by euthanasia or by using hand-laid cyanide baits, and necropsied for TB. Traps and poison baits were deployed in areas of preferred possum habitat, such as gullies and at the edges of patches of scrub. Animal size and dental wear patterns [44] were used to class possums as immature yearlings or as adults (the latter > 1 year of age). Body condition was classed subjectively according to a number of features, including general appearance and coat condition (assessed during palpation for gross TB lesions) and the amount of kidney fat (assessed during necropsies). Necropsies generally followed the standard possum TB necropsy procedure outlined in S1 File, which included routine mycobacteriological culture of pooled predilection site tissues of each animal necropsied. In addition to any pathologies that were indicative of clinical TB (classed ‘typical’), any that were either suggestive of TB (classed ‘equivocal’) or were non-suggestive but occurred at a predilection site (peripheral or mesenteric LNs), were also excised from the parent tissue, frozen and then submitted for *M*. *bovis* culture individually. All *ex vivo* tissue samples were transported to a laboratory freezer and stored frozen at -20°C within 24 hours of collection. Culture for confirmation of *M*. *bovis* infection was undertaken on homogenates prepared from thawed samples by the National Centre for Biosecurity and Infectious Diseases (AgResearch, Upper Hutt, New Zealand).

All possums were necropsied but only adult possums were considered in the assessment of vaccine efficacy, since juveniles had likely been born (or newly weaned) after vaccine deployment and therefore were unlikely to have had exposure to the vaccine.

### Data accessibility

Data presented in this study are publicly accessible at http://datastore.landcareresearch.co.nz/dataset/data-from-nugent-yockney-et-al-aerial-bcg-vaccine-trial-possums. The data sets refer to 1] fate-of-bait data from the comparative choice baiting trial, 2] CC and fate-of-bait data from the single-choice baiting trial, 3] immunological data (as stimulation indices in response to PPD-B) from the 2-month post-vaccination study, 4] necropsy and culture data from wildlife caught or shot in the area prior to vaccine deployment, 5] necropsy data from the 13-month post-vaccination study (concerning possum demographics, block designation and treatment applied, lesion occurrence, and *M*. *bovis* culture results).

## Results

### Pre-trial possum population density and TB levels in resident wildlife

In October 2010, a trap-catch index of 3.3% (n = 368 trap nights) was recorded in the pilot trial area, indicating that possum density over the whole area was very low (< 0.6 possums/ha). At such low densities, estimates are imprecise [36]; however, by way of comparison, much higher trap-catch rates of 28–29% were recorded by Rouco et al. [30] in similar habitat where the measured possum densities subsequently proved to be in the range 0.4–0.7 /ha.

Surveys conducted 6–24 months before the main vaccine field trial confirmed substantial TB presence in wildlife in the study area, with culture-confirmed TB lesions recorded in 67.0% (95% CI, 57–77) of a total of 91 pigs (Table 2). Of 98 possums surveyed, five had typical macroscopic lesions, representing a gross TB prevalence of 5.1% (95% CI, 0.7–9.5). There was also substantial sub-clinical infection in the sample, with a further 5 cases of *M*. *bovis* infection detected among 51 of the 93 possums that had no visible lesions (NVL/culture-positive; 9.8%).

**Table 2:** Details of possums and pigs necropsied from wildlife TB surveys conducted on Muzzle Station/Clarence Reserve between spring 2009 to autumn 2011 (final surveillance was 6 months prior to vaccine deployment).

	No. necrop-sied	Males, females	Yearling, adult	Culture-confirmed TB lesions	No. of NVL cases cultured	No. of NVL cases culture positive
Possums	98	59m, 39f	23y, 72a *	5 (5.1%)	51	5
Pigs	91	48m, 40f *	23y, 68a	61 (67.0%)	30	0

* Sex not recorded for 3 pigs, age status not recorded for 3 possums.

Note: of 93 NVL possums, only a sub-sample of 55% (n = 51) were submitted for mycobacteriologial culture to detect sub-clinical *M*. *bovis* infection.

### Pre-trial pilot studies: bait-delivery and wildlife interactions

In the first assessment of free-choice uptake of sachet-held bait, possums were confirmed as having visited bait sites (by bite marks on CCs) on 12 separate occasions over three nights at 8 of the 33 sites where rhodamine-dyed baits had been set. Eight adult possums (3 males, 5 females) were then trapped in the vicinity over the next two nights, all of which had been marked by rhodamine-B. Two of the female possums that were trapped were carrying dependent juveniles, neither of which bore rhodamine marking.

In the pilot sowing trial, 186 baits were successfully deployed from the helicopter, equating to a delivery density to 3.7 baits/ha. Ground searches along the flight paths located 101 sachets within line-of-sight of the paths—98 were still intact, one was torn but still contained the bait, and two were torn with the bait removed. Fourteen sachets were found trapped in thorny shrub vegetation 0.3–2.0m off the ground. The number of intact sachets found equates to a confirmed presence of least 2.02 visually-locatable baits/ha for the second night after aerial sowing. The difference between the density of baits sown and the number we found after the first night is possibly not only because some baits were simply not found, but because some may have been moved away from the flight path by possums.

By re-searching over the flight deployment area along transects set at 90 degrees to the original flight paths, 57 aerially-delivered sachet baits were located and marked by waypoints for the next pilot trial. Over 3 subsequent nights of exposure, 40% of these sites in total had the bait either confirmed eaten (14%) or the sachet and bait removed from the site (26%) (Table 3). This was higher than for the cereal baits subsequently deployed in the same area, for which 10% were confirmed eaten and 9% had the sachet and bait removed after 3 nights.

**Table 3:** Summary of results from fate-of-bait pilot trials, to determine the contact and removal rates of flavoured lipid baits from habitat on Muzzle Station/Clarence Reserve over 3–7 day periods and to identify the likely wildlife species involved.

	Baiting characteristics			Proportional fate of baits and chew card indication of possum activity			
Trial type	No. baits	Bait spacing	Nights baits available	Not touched	Eaten *in situ*, sachet remaining	Destroyed or removed from site	Proportion of removals or consumptions CC+
Comparative removal							
Lipid bait	57	~88m*^1^	3	60%	14%	26%	N/A
Commercial possum bait	146	33m	3	81%	10%	9%	N/A
Single choice							
Lipid bait (with attendant CC)	316	50m	7	38%	31%	31%	53%*^2^

*^1^ refers to 57 lipid baits located and marked by waypoints over 5000 m of transect lines for the comparative bait removal study, averaging one bait approximately every 88 m of the transects.

*^2^ refers to the percentage of those sites where there was indication of wildlife contact with the lipid bait (i.e. bait had been removed, destroyed or eaten) that also showed possum activity in the immediate proximity, via dental indentations on an attendant chewcard (CC).

In the pilot ground-based deployment trial conducted in the main trial blocks, examination of CCs after 7 days indicated animal bite marks at 143 (45%) of the 316 sites where CCs had been placed next to sachet baits (Table 3). Overall, approximately one-third (113) of the 316 sites were definitely visited by possums, with visits from rodents, mustelids or hedgehogs making up the remainder (as identified by species-specific bite marks on the CCs). Another 71 sites had no CC bite marks but had some or all lipid bait consumed or removed from the site. Of the sites definitely visited by possums, 105 (93%) had some or all lipid consumed or removed from the site, with partial (1) or complete (85) consumption of the lipid at 86 sites and complete removal (including the sachet) at 19 (18%) sites. In contrast, at the 92 other ‘no possum presence detected’ sites with indications of some lipid consumption or removal, 86% of sachet baits were completely removed.

### Vaccine deployment

Sachets baits were successfully deployed by aerial delivery from a helicopter, as judged by a ground-based observer (GN) positioned under the flightpaths who was readily able to visually track samples of baits (15–20) as they fell. The day of deployment was clear and still with ground frost, and shady south-facing slopes remained frosty throughout the day, so the lipid remained solid except that for a few of the baits which landed on ground in full sun on north-facing slopes, where the lipid matrix had begun to melt by late afternoon.

### Post-vaccination immune reactivity

**Fig 2:**
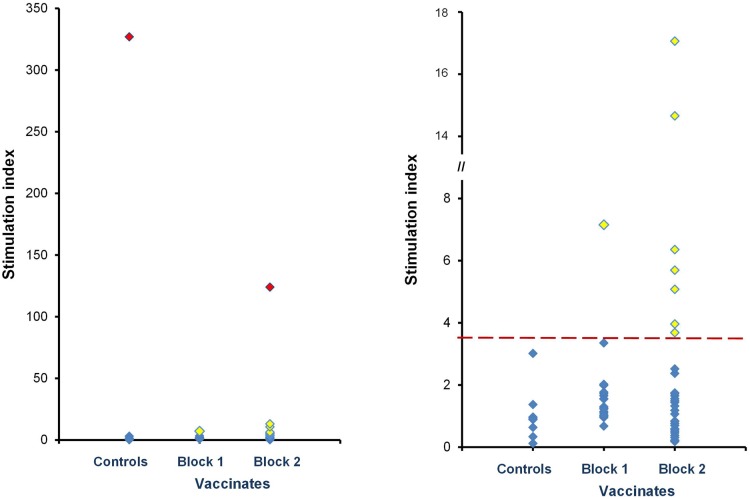
Summary of LPA responses to PPD-B—stimulation among possums captured from a non-vaccinated block and two vaccine blocks, two months following aerial vaccine deployment. Diamond symbols indicate LPA responses to PPD-B in terms of a stimulation index. Left-fig, data from 61 possums, highlighting 2 animals that were excluded from subsequent analyses on the basis of likely pre-existing *M*. *bovis* infection (red symbols). Right-fig, data from 59 possums (excluding the 2 likely infected cases) re-plotted on a reduced scale graph. Yellow symbols represent responses of individual animals that were greater than the 99 percentile distribution of the control values (i.e. > control mean + 3 standard deviations, indicated by the red dashed line).

Blood samples were collected for LPA from 9 possums trapped from one non-vaccinated block, and 52 possums trapped from the two vaccine-treatment blocks. The majority of blood samples (59/61) had SI values between 0.1 and 17.1; however, two animals (one from the non-vaccinated block and one from the treatment blocks) had extremely high SI values of 124 and 327. Given such values most likely reflect responses to virulent *M*. *bovis* infection [26], these individuals were excluded from subsequent analyses (Fig 2). Among the remaining 59 possums, 8/51 animals from vaccine blocks showed immune reactivity with an SI > 3.65 suggestive of a positive post-vaccination response (16% response rate; 95% CI 6–26%).

### Field study of the effect of aerial vaccine deployment on TB parameters

At trial completion, the overall prevalence of *M*. *bovis* infection in unvaccinated areas was 5.8% (95% CI, 1.2–10.3) among a total of 104 adult and juvenile possums captured and euthanized.

In total, 181 adult possums (89 from non-vaccinated blocks and 92 from vaccine blocks) were killed and necropsied in October 2012, 13 months after vaccine deployment, with this sample including no artificially infected animals. Of these, six had culture-confirmed *M*. *bovis* (five animals with typical or equivocal lesions, and one animal that was NVL), comprising five from the non-vaccinated blocks and one from the vaccine blocks (Table 4). The estimated prevalence of *M*. *bovis* infection in adult possums in the unvaccinated blocks (mean 5.6%, 95% CI 0.7–10.5, n = 89) was thus over five times higher than in the vaccinated blocks (1.1%, 0–3.3%, n = 92). Pooled by treatment, the difference is marginally significant (P = 0.098; one-tailed Fisher’s exact test).

**Table 4:** Prevalence of culture-confirmed *M*. *bovis* infection and gross pathology among 181 adult (> 1 year old) possums captured from vaccinated and non-vaccinated blocks.

	No. possums	No. with infection (lesions)	Infection prevalence, % (95%CI)	Gross lesion sites (by each culture-positive individual animal)
Non-vaccine blocks:				
1	38	3 (2)	7.9 (0–16.6)	1] NVL (female possum, 3.1kg, good condition) 2] Lung pathology only (female possum, 1.6kg, poor condition) 3] Lung pathology only (female possum, 3.1kg, average condition)
2	51	2 (2)	3.9 (0–9.4)	4] Peripheral LN lesions* and lung pathology (male possum, 1.6kg, poor condition) 5] Liver lesions and lung pathology (female possum, 3.3kg, good condition)
*All non-vaccinates*	*89 (51F*, *…*..*38M)*	*5 (4)*	*5*.*6 (0*.*7–10*.*5)*	
Vaccine blocks:				
1	54	0	0	N/A
2	38	1 (1)	2.6 (0–8.0)	1] Peripheral LN lesions*, MLN lesions and lung pathology (male possum, 1.9kg, very poor condition)
*All vaccinates*	*92 (53F*, *……39M)*	*1 (1)*	*1*.*1 (0–3*.*2)*	

*Severe peripheral site pathology, including draining sinus tracts from unilateral superficial axillary region. NVL, no visible lesions but *M*. *bovis* culture-positive predilection site tissues; MLN, mesenteric lymph nodes.

## Discussion

We sought to develop a practical tool for reducing or eliminating TB from a wildlife host living within remote areas of difficult terrain where the usual approach (lethal possum control) is not feasible or not socially-acceptable. We considered that the successful development and laboratory testing of the lipid matrix as an effective carrier for live BCG [45], its ability to be flavoured and made attractive and palatable to possums [40], and the vaccine’s indicated 95% efficacy in preventing natural *M*. *bovis* infection among free-ranging (but hand-vaccinated) possums [26], together provided sufficient *a priori* indication that the vaccine would work in the field, if it could be effectively delivered to (and freely consumed by) wild possums. This would avoid the impractical requirement of having to catch wild animals first to vaccinate them by hand [26, 46–50]. As a vehicle for delivering a free-choice vaccine, aerial distribution was the obvious choice for the particular disease control context we focussed on.

In North America and in Western Europe, aerial distribution of free-choice oral vaccines has been used to vaccinate mesocarnivores against sylvatic rabies and to vaccinate wild boar against classical swine fever (CSF) virus [51]. In line with those examples of aerial vaccine delivery, our studies first confirmed that aerial delivery of sachet baits containing live BCG was likewise technically feasible, and straightforward given the technological developments that have already taken place for aerial delivery of toxic baits in pest control in New Zealand [15,42]. The desired ultimate outcome from our studies (demonstration of a reduction in TB prevalence in possums by aerial vaccination) appears to have possibly been achieved since the measured *M*. *bovis* infection prevalence 13 months after vaccine deployment was markedly lower in the vaccinated blocks. Our sample sizes were too small to confirm the significance of protection with high statistical power, but, if not a chance effect, our result suggests a vaccine protective efficacy of 81%.

Retrospective power-based sample size estimates have indicated that—with the low endemic prevalence of TB among possums in the NSIHC—further or repeat vaccine trials in this area would require greater effort if they are to stand a chance of achieving a result with strong statistical validity, involving in excess of 350 adult possums (which would require the trapping of ~400 possums in total). That was not achievable with the trapping resources available to our studies largely because, with hindsight, the density of possums in this area was far lower than anticipated, although we cannot state with precision how low. At the time the study was instigated, the 3.3% trap-catch rate in the pilot trial area, along with previous survey data [28], was assumed to reflect a density of ~0.6 possums/ha; but that estimation was obtained using a conversion rate based on computer simulation of possum trappability in forest environments [36] where their home ranges are usually only a few hectares [52]. Since then, new studies have shown possums in semi-arid areas similar to our study area have far larger home ranges (average of about 22 ha [31]) and are probably therefore more readily trappable, with Rouco et al. [30] recording live-trap trap-catch rates of over 20% in a similar semi-arid area, even though that area had a measured possum density averaging only 0.5 possums/ha overall. Those facts, along with very recent and more precise mark-recapture data from Muzzle Station indicating a possum population density of 0.18/ha [53], together suggest that true possum densities in trial blocks in our study are likely to have been below 0.6 possums/ha and possibly even below 0.2/ha—i.e. the two ~1000 ha vaccine blocks could have contained fewer than 400 possums overall (and the 1360 ha that was actually baited, fewer than 270 possums). Hence, we conclude retrospectively that a robust determination of vaccine efficacy was not likely achievable using the trapping effort (and consequent sample sizes) achieved in our studies, even allowing for the fact that we supplemented the local force of infection by releasing artificially-infected possums.

Nevertheless, the statistically weak evidence of a vaccine effect that we recorded here is supported by the blood-testing results. Definitive blood tests to detect positive BCG vaccine responses, and to distinguish these from responses observed to virulent *M*. *bovis* infection, are not yet available or reliable for use in free-ranging wildlife [20], however we are confident that some of the aerially-delivered BCG vaccine did reach possums in our studies. Probable cases of true cell-mediated immune reactivity, i.e. positive LPA responses to PPD-B, were recorded in 16% of the possums sampled from vaccine blocks two months post vaccine-deployment, but not in the no-vaccine blocks. This represents a minimum likely response rate because, due to the stress effects on the immune response associated with live capture [54], it is known that not all vaccinated possums in the field are likely to respond positively in an LPA, even with assured vaccine uptake. For example, Tompkins *et al*. [26] reported positive vaccine responses in just 35% of wild possums that had previously been trapped and hand-vaccinated (and thus had an assured vaccine uptake rate of 100%). In that trial, the 35% positive vaccine LPA response was correlated to a subsequent protective efficacy of 95% for the lipid-BCG vaccine against *M*. *bovis* infection. The disparity between the 16% LPA positives in our trial and the 35% recorded in confirmed vaccinated possums by Tompkins *et al*. [26] suggests that, under free-choice conditions, only about half the possums in our trial may have been successfully vaccinated. Prior epidemiological modelling by Barlow [55] has suggested that an annual vaccination rate of 70% would be required to reduce the TB prevalence in a typical possum population to 90% of its starting value within 5 years. If the vaccine coverage rates here were, in actuality, not far short of that (i.e. ~50%), there are several potential explanations for why a higher vaccination rate was not achieved.

First, the baiting rate could simply have been too low for all possums to encounter vaccine. That is unlikely because our deployment rate (= 300 BCG baits/ km^2^) was substantially higher than maximum deployment rates of up to 150 baits/km^2^ used in aerial rabies vaccination campaigns [56–60], while modelling probabilities of possum encounters with poison baits or traps in this type of habitat [32] leaves little doubt that possums would have encountered the BCG baits. Second, the supply of baits could have been depleted before all possums were able to encounter them. That also seems unlikely, given that in pilot trials a third of hand-placed baits remained undisturbed after 7 nights. Thirdly, the BCG baits may not have been sufficiently palatable to achieve a high uptake rate. However, a previous study [40] showed 85–100% of possums had consumed a similar bait when deployed in forested habitat, and there were few examples of the lipid baits only being partly consumed in our bait acceptance trials. Lastly, there is the possibility of a decline in vaccine viability over time in face of quite wide variation between day and night temperatures, which is typical for the NSIHC area. For this latter consideration we have no data to assess whether this was a problem, but if it was, it is likely to be far less significant if vaccination in the future is conducted under cool temperate winter conditions in lower elevation forested areas that are likely to be the main targets for aerial vaccination.

Overall, we conclude that aerial delivery of a sachet-held TB vaccine by simply dropping bait from a helicopter flown at low speeds is feasible, and could be readily achieved in steep difficult terrain where other options for TB control in possums are not viable. We further show evidence, for the first time, of at least some free choice uptake of a live oral vaccine by wild possums, with weak evidence that this subsequently resulted in a protective effect. The 81% vaccine efficacy calculated from our results compares well to the two previous BCG-based vaccine studies in possums where 69% and 95% efficacy was recorded, respectively [26,46]. Those previous trials were conducted among possum populations at higher density in more typical forested habitat and relied on hand-vaccination of captured possums, using BCG administered to the nasofacial mucosae [46] or BCG administered to the oral cavity in the lipid matrix [26]. Importantly, estimated TB levels among non-treatment possums in those previous trials were >8% [46] and >16% [26] compared to <6% among possums in the present study, even though we had attempted to increase the local force of infection through the deliberate release of *M*. *bovis*-infected animals [61].

More broadly, the available laboratory, domestic and wild animal studies using oral-delivery lipid-formulated BCG as a vaccine against *M*. *bovis* in a variety of species [21,25,26,45,62–66] show unequivocally that this vaccine can, if properly delivered, provide measurable protection against tuberculosis. Thus while we are uncertain as to whether we achieved a sufficiently useful level of protection here, that uncertainty relates mainly to the level of uptake achieved rather than vaccine efficacy *per se*. Mitigating the possible problems with bait uptake and viability should be straightforward. Other lures or flavourings could be developed, as research using the lipid vaccine matrix with different flavourings and attractants for other wildlife species has indicated [67]. Alternatively, baiting could be conducted when natural food is scarce and in seasons and habitats with minimal day-night variation in temperature. Overall, none of these problems appears to be intractable, and we suggest that, with some refinement, a repeat of this trial would have a high prospect of successfully reducing TB prevalence. A practical challenge to such a repeat would be finding a suitable site, as the inaccessible areas in which vaccination might be the only viable TB management option are (by definition) unworkable. There are now few readily accessible areas in New Zealand where TB remains at high prevalence in possums [68]. That reflects success in greatly reducing TB levels nationally through use of established depopulation tools (trapping and poisoning) [11], an achievement that in itself undermines the perceived need for a tool such as vaccination. Those effective and (comparatively) low-cost tools currently have strong governmental support [69] despite some public antipathy in New Zealand to the widespread use of aerially-delivered poison baits [16]. Thus, although simulation modelling indicates that oral vaccination of possums would potentially be an effective and affordable TB control strategy (particularly when used in conjunction with methods that reduce possum densities [24]), further development of aerial vaccination of possums appears likely to occur only if there are significant inaccessible areas where aerial poisoning is not possible or permitted. Such areas do exist, but to date they have not been an important impediment to New Zealand’s TB management objectives because historical objectives did not require complete eradication of the disease. In 2016, however, national eradication of bovine TB (rather than control) became the goal [70] so such difficult areas can no longer be left unmanaged. We suggest that aerial BCG vaccination could, potentially, be quickly developed into an operational tool that would enable managers to eliminate TB from such areas with much reduced need for repeated application of toxicants.

## Supporting Information

S1 File Necropsy procedure for detecting TB and *M*. *bovis* infection in possums. (DOCX)
