# Supplementary material for: Field Trial of an Aerially-Distributed Tuberculosis Vaccine in a Low-Density Wildlife Population of Brushtail Possums (Trichosurus vulpecula)
Source: PLoS One. 2016 Nov 28;11(11):e0167144. doi: 10.1371/journal.pone.0167144 (PMC5125682; doi:10.1371/journal.pone.0167144)
Supplement: S1 File — (DOCX) [file pone.0167144.s001.docx]

**Necropsy procedure for detecting TB and *M. bovis* infection in possums**

Diagnosis of *M. bovis* infection was based on physical examination of the carcass, necropsy, fine dissection of predilection-site tissues and mycobacteriological culture of pooled lymph nodes (LNs) as follows:

1] Visual inspection of the carcass for draining sinuses indicative of fulminating TB; palpation of the carcass (peripheral sites, abdomen, thorax, mandibular region) for swellings suggestive of advanced TB.

2] Necropsy and macroscopic examination of lungs, liver, spleen for gross lesions suggestive of advanced TB.

3] Excision of superficial and deep axillary LNs, inguinal LNs and mesenteric LNs; and fine (~2mm interval) serial incision for lesions suggestive of clinical TB.

4] Any typical or equivocal lesions from above are submitted for mycobacteriological culture, with outgrowths subsequently confirmed as *M. bovis.*

5] In the case of no visible lesions (NVL), excised superficial and deep axillary LNs, inguinal LNs and mesenteric LNs are macerated with a sterile scalpel blade; approximately 1 g of each NVL macerate is archived at -20C while a further ~1 g of the NVL macerate is added to an accumulative tissue pool; the pool is continued until it comprises tissues from 4 or 10 animals, then it is submitted for *M. bovis* culture.

6] In the case that any NVL pool sample from 5] above proves *M. bovis* culture-positive, each of the 4 or 10 contributing individual NVL macerate samples is thawed from the archive and submitted for retrospective (individual) *M. bovis* culture.

The above procedure has a combined diagnostic sensitivity for detecting *M. bovis* infection in possums of 95%.
